# Lung Disease in Rheumatic Disorders

**DOI:** 10.31138/mjr.30.3.147

**Published:** 2019-09-30

**Authors:** Clive Kelly

**Affiliations:** University of Newcastle upon Tyne, Newcastle, United Kingdom

**Keywords:** rheumatoid arthritis, interstitial lung disease, bronchiectasis, scleroderma, Sjögren’s

## INTRODUCTION

Rheumatology and Chest Medicine remain arguably the last two bastions of clinical medicine. Admittedly, both have benefitted from scientific insights as to processes and pathology that underpin their clinical manifestations, but a thorough history and careful examination remain essential prerequisites to the successful diagnosis and management of most of the disorders encompassed by each speciality. Hence, the overlap between these two specialities also remains an area of essentially clinical based assessment. However, the combination of newer imaging techniques and exciting novel therapeutic agents has begun to impact favourably on both the clinical manifestations of these disorders, and their outcome. As our clinical insights have been augmented by experience and a growing evidence base to guide diagnosis and management, there have been significant measurable improvements in both the quantity and quality of life associated with the development of lung disorders in patients with rheumatic disease. Even more exciting is the prospect that these benefits may be applicable to those with the lung diseases, even in the absence of a related rheumatic disorder. The recognition of the role of antibody-marked or sometimes mediated disease has been an important factor in recognising the potential to apply the lessons learned from the treatment of lung disorders in rheumatic disease to the lung diseases in isolation.

This review article will discuss the lung conditions most commonly associated with rheumatic disorders and will focus on the following areas: interstitial lung disease, bronchiectasis, pulmonary hypertension and pleural effusions. Likewise, we will discuss the rheumatic disorders associated with these conditions which include: rheumatoid arthritis, systemic sclerosis, systemic lupus erythematosus, primary Sjögren’s and mixed connective tissue disease.

## PLEURAL EFFUSIONS

Pleural effusions have become relatively rare in the context of many rheumatic disorders in the last decade throughout Europe. This is often thought to relate to the earlier and more effective introduction of disease modifying antirheumatic drugs (DMARDs) and biologics but may be in part due to a reduction in the natural aggression of the underlying disease process. Effusions occur most often in rheumatoid arthritis (RA)^1^ and systemic lupus erythematosus (SLE) and are most often left-sided. They may coexist with a pericardial effusion and, in extreme cases, may occur when pericardial tamponade causes fluid to leak into the adjacent pleural space under increasing pressure, either spontaneously or as a consequence of the surgical procedure of pericardial fenestration, when a window in the pericardial sac is created to permit such drainage. Such a development was not uncommon until the end of the last century, and fenestration was often considered lifesaving if a pericardial effusion recurred after initial simple pericardiocentesis had failed.^3^

Pleural and pericardial effusions now more commonly coexist in the context of SLE than RA, and such serositis may be a presenting feature of the disease in some cases.^4^ As an early feature, it is important for clinicians to be aware of the potential association with other systemic manifestations of SLE such as nephritis and even encephalitis which can develop with alarming rapidity in systemic disease, especially in young women of African origin.^5^ Hence, the clinical signs of a pleural effusion should trigger a careful examination for signs of pericardial tamponade by inspection of the jugular venous pulse (elevated) and brachial arterial pressure (reduced, especially during inspiration) which may be a more life-threatening cause of the dyspnoea these patients usually develop. If suspected on clinical grounds, an Echocardiogram is an essential and urgent investigation to complement the chest radiograph and electrocardiogram that should be mandatory in all such patients. Even where a pleural effusion exists in isolation, simple aspiration under local anaesthesia may not be sufficient to offer permanent relief of symptoms. Recurrence is common, especially in the presence of markers of active disease, and investigations to confirm that the effusion is related to the rheumatic disorder are necessary.

Pleural fluid should therefore be sent after aspiration for laboratory analysis. Protein levels over 30 g/L indicate inflammatory disease in the form of an exudate, and an elevation in both LDH and rheumatoid factor may be reported. On cellular examination, lymphocytes are usually predominant in the acute setting although neutrophils may influx into more chronic effusions when the pleura can become progressively thickened.^6^ Glucose levels drop under such circumstances, and occasionally secondary infection occurs, forming an empyema. This is important to exclude, as is tuberculous infection, so fluid should also be sent for bacteriology especially if the patient is pyrexial, systemically unwell or immunosuppressed. Once infection is excluded, therapeutic intervention with DMARDs, often combined with a short course of oral prednisone, is usually indicated. In those patients with resistant or especially active disease, biologic therapy may be commenced as an early intervention. Although this often improves pleural effusions, cases of exacerbation of pericardial disease have been recorded and debate around the efficacy of this approach continues.^7^

## PULMONARY HYPERTENSION

Although primary pulmonary hypertension remains a relatively rare condition, the development of raised pulmonary artery pressure occurs in certain connective tissue diseases much more commonly than by chance.^8^ Mechanisms differ however between the two commonest secondary forms of the disease. In patients with systemic sclerosis (scleroderma), a gradual onset of pulmonary hypertension ensues in up to 40% of patients.^9^ Symptoms of fatigue and breathlessness are often non-specific and many patients initially attribute these to their underlying disease. As the disease process advances, then right ventricular failure often develops with progressive peripheral oedema. In limited cutaneous scleroderma (previously referred to as CREST) the cells lining the intima of the pulmonary vascular are damaged and the stromal tissue becomes inflamed with resulting narrowing of the vessels.^10^ By contrast, in patients with diffuse cutaneous disease, the development of raised pulmonary pressure is typically a consequence of progressive interstitial lung disease (ILD). In patients with SLE, pulmonary hypertension is more likely to result from thrombo-occlusive disease in the pulmonary arteries. This in turn may develop either as a consequence of peripheral venous thrombosis leading to pulmonary emboli, or as a result of the presence of anti-cardiolipin (ACl) or anti-phospholipid (APL) antibody causing in-situ thrombosis within the peripheral arterioles and sometimes central pulmonary arteries.^11^

Patterns of autoantibodies may be useful in defining the risk of pulmonary hypertension in scleroderma. Patients with positive anti-centromere antibody are at considerable risk of primary vascular disease, while those with Scl70 antibodies are at greater risk of secondary pulmonary hypertension due to the development of ILD.^12^ Investigations are essential in diagnosing this process, as it may be difficult to make on purely clinical grounds. Pulmonary function tests will demonstrate a significant reduction in gas transfer, while lung volumes may be relatively preserved in the absence of ILD. A chest radiograph may be normal or show pruning of pulmonary vasculature, while CT pulmonary angiography will show pulmonary emboli where present in segmental arteries and sometimes in smaller vessels. Electrocardiography will often show right heart strain, sometimes with T wave inversion and tachycardia. In the presence of emboli, an S1Q3T3 pattern is often observed, sometimes with right bundle branch block. Echocardiography will demonstrate right ventricular dilatation as it has limited potential for hypertrophy and right ventricular overload is seen in the presence of clinical oedema.^13^ D dimers will be elevated in the presence of pulmonary emboli or in-situ thrombosis.

Treatment depends on the cause.^14^ Lifelong anticoagulation is required for patients with evidence of recurrent pulmonary embolism and is mandatory in the presence of associated APL antibodies. In certain cases, surgical treatment with endarterectomy to remove accumulated thrombosis attached to the arterial wall is needed. For those with limited scleroderma and primary vascular disease, treatment with a range of medical options is recommended. Vasodilators are usually commenced in the form of calcium channel antagonists initially, with endothelin receptor antagonists, phosphodiesterase 5 inhibitors and prostanoids in either intravenous or nebulised form. Newer therapies including selexipag and nitric oxide offer promising future options. For patients who develop decompensated right heart failure, oxygen and diuretics, together with anticoagulation, is usually required.

## BRONCHIECTASIS

Bronchiectasis (BR) is the anatomical distortion of conducting airways that results in chronic cough, sputum production and recurrent infection.^15^ RA patients have a much higher prevalence of symptomatic bronchiectasis compared with the general population.^16^ The prevalence of anatomical changes consistent with bronchiectasis on HRCT studies is reported in up to 30%,^17,18^ illustrating that many RA patients may have subclinical structural bronchiectasis. This may either precede or complicate the development of articular disease and is strongly related to females who exhibit high titres of anti-cyclic citrullinated peptide (CCP) antibodies. We have demonstrated anti-CCP positivity in 94% and rheumatoid factor (RF) positivity in 97% of patients with RA-BR, which is significantly higher than would normally be seen in patients with RA alone.^19^ The mechanism is unknown, although RA-BR patients are frequently never smokers,^20^ hence a group that we would not expect to be at high risk of ACPA-positive RA. An alternative trigger may drive the development of ACPA-positive RA in this group. Given the high prevalence of anatomical structural changes consistent with bronchiectasis on HRCT of RA patients, it is possible that this trigger is in the lung.

Patients usually present with cough productive of coloured sputum, and often demonstrate evidence of recurrent chest infections. Clinical examination typically shows lung crackles at one or both bases which become increasingly persistent and coarse. In advanced disease bi-basal coarse lung crackles with or without finger clubbing, make differentiation from ILD difficult without imaging. In addition, compared with ethnicity-matched control populations with RA alone, an association with HLA-DRB1*0405 has been identified in a cohort of Japanese RA-BR patients^21^ and an association with the shared epitope (SE) allele HLA-DRB1*0401 has been identified in a cohort of French RA-BR patients.^65^ This strengthens the hypothesis that the presence of bronchiectasis with or without recurrent infection may drive the development of RA autoantibodies in susceptible individuals (*HLA-DRB1* SE carriers).

Patients with RA and bronchiectasis often have active articular disease with Disease Activity Scores (DAS 28) elevated above 5, which are resistant to standard disease modifying drug (DMARD) therapy. In this clinical situation biologic therapy may be required. Clinicians are often concerned at the potential for exacerbating the risk of chest infections with the use of anti-tumour necrosis factor (TNF) therapies, but rituximab appears both safe and effective in most cases and may even improve the outcome of the lung disease itself.^23^

## INTERSTITIAL LUNG DISEASE

Interstitial lung disease (ILD) is common in a number of rheumatic disorders, with the strongest association being found in systemic sclerosis (scleroderma). In scleroderma patients with diffuse cutaneous disease, ILD has been reported in over 40% of patients and is the cause of death in half of these.^24^

**Figure 1.**
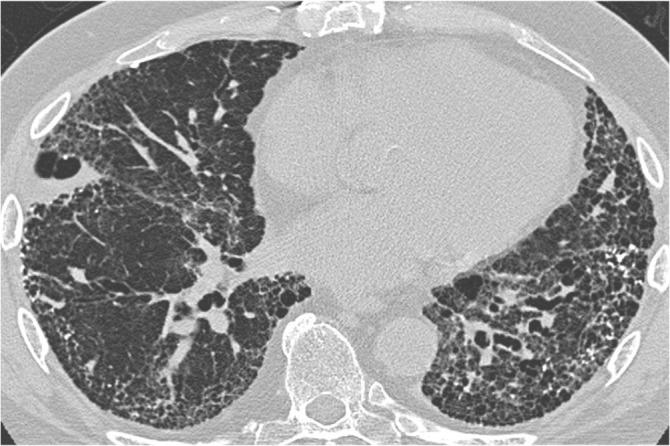
High resolution computed tomography of the lungs in a patient with RA-ILD, demonstrating changes of usual interstitial pneumonia with bibasal fibrosis and early honeycombing.

ILD is also well recognised in RA which is a lot commoner than scleroderma and therefore accounts a greater total number of cases. ILD has been recognised in RA for over 50 years and has been reported in up to 40% established RA patients at post-mortem.^25^ Although, it is evident in up to 25% of RA patients on HRCT (*Figure 1*),^26^ it is less commonly clinically significant, with a clinical prevalence of around 5%. The lifetime risk of ILD in RA patients was calculated at 7.7% by Bongartz et al.^27^ with a hazard ratio of HR of 9 and a relative risk of 3 for death. Until recently, the mean survival from diagnosis for RA-ILD was just 2.6 years, although that may now be improving with newer therapies. RA-ILD remains the only complication of RA presently reported to be increasing in frequency.^28^ ILD in RA is associated with male sex, smoking and the presence of strongly positive anti-CCP antibodies. We theorise that it is a consequence of citrullination occurring as a result of activation of B cells in lungs or gums.^29^

ILD is recognised in in other rheumatic diseases. In systemic lupus erythematosus (SLE), true pulmonary fibrosis is not very common. Diffuse alveolar damage is the most serious form of lung involvement and is often fatal, especially with haemorrhage.^30^ This may coexist with pulmonary embolism. True ILD can be early and rapidly progressive in genetically susceptible populations, especially in those of Afro-Caribbean origin. In Caucasian populations, true inflammatory or fibrotic alveolar disease affects less than 5% of patients. In primary Sjogren’s syndrome, both lymphoid interstitial pneumonia and cryptogenic organising pneumonia are well described. Both have relatively good natural history and are usually responsive to oral prednisone.^31^ Neither is likely to progress to fibrosis if identified and treated early enough. The prevalence of inflammatory ILD in primary Sjögren’s syndrome is estimated at 30% but with low conversion rates to pulmonary fibrosis.^31^ In patients with mixed connective tissue disease (MCTD), multi organ involvement is common but the lungs not usually severely affected. Non-specific interstitial pneumonia (NSIP) is the usual common pattern with usual interstitial pneumonia (UIP) before relatively rare. Patients often appear responsive to use of mycophenolate and fibrosis relatively rare and late in onset.

Interstitial pneumonitis with auto-immune features (IPAF) is a relatively recently described entity.^32^ This term was proposed to describe the presence of a combination of features from any two of three domains: a clinical domain, consisting of specific extra-thoracic features; a serological domain, consisting of specific autoantibodies; and a morphological domain, consisting of specific chest imaging, histopathological or pulmonary physiological features. Oldham et al.^33^ retrospectively applied IPAF criteria to a cohort of 422 patients diagnosed with IIP, interstitial pneumonia associated with undifferentiated connective tissue disease (UCTD) or unclassifiable ILD. 144 met IPAF criteria. Morphological and serological domain criteria were both satisfied in over three-quarters of IPAF patients. Mortality in patients meeting IPAF criteria was lower than in patients with idiopathic pulmonary fibrosis (IPF), and higher than in patients with CTD-ILD. Those IPAF patients with UIP (n=98) had same adverse prognosis as IPF while those with non-UIP pattern (n=46) had the same improved prognosis associated with CTD-ILD.

The burden of ILD among patients with rheumatic disease is high, both in terms of mortality and associated morbidity, with symptoms reducing quality of life, exercise tolerance and employment prospects, while increasing costs of supportive care in both hospital and the community.

Using HRCT to define the pattern of lung involvement is an essential aspect of the diagnostic assessment. Usual interstitial pneumonia (UIP) accounts for the majority of cases in RA^34^ and this carries a worse prognosis than non-specific interstitial pneumonia (NSIP) which is reported to be present in about a quarter of cases. These are generally more responsive to early therapy. Both cryptogenic organising pneumonia (COP) and overlap syndromes are well recognised but relatively rare, each accounting for about 5% of total cases. The respiratory mortality associated with UIP is approximately four times that reported in NSIP.^35^ Another important factor determined by HRCT is the extent of the lung disease, with more than 20% of lung involved indicating extensive disease. The presence of extensive over limited disease also doubles mortality so that an RA patient with extensive UIP has an approximately eight-fold increased risk of dying over the ensuing year than a patient with limited NSIP.^35^ Lung function tests show a reduction in total lung volumes and forced vital capacity as the lungs shrink with progressive fibrosis. This reduction accelerates with increasing pulmonary fibrosis. Gas transfer falls rather more quickly and dramatically, as alveolar structure and function are diminished by progressive distortion from increasing fibrosis

Predictors of progression on HRCT include disease subtype (UIP fares worse than NSIP), disease extent (limited disease does better than extensive), while lung function predictors of poor prognosis include a baseline vital capacity below 60%, and a reduction in baseline gas transfer to 50%. Monitoring of progression of ILD should be considered using lung function testing (spirometry and gas transfer) every 6 months and HRCT every two years.

## TREATMENT OF RA-ILD

There have been significant changes in the survival associated with RA-ILD. *Table 1* shows data from the British Rheumatoid InterstitiaL Lung (BRILL) network which studied outcome over 25 years in 290 patients with RAILD. This demonstrated progressively increasing survival by quartile over the last 25 years, associated with lower risk of a respiratory death. This is in part explained by our evolving therapeutic approach to the management of ILD in RA and other rheumatic disorders. An important determent is the subtype of disease as assessed by HRCT. Those with COP (eg, primary Sjögren’s syndrome) usually respond well to oral steroids in modest doses, while patients showing an NSIP pattern (eg, scleroderma) generally have significant inflammatory alveolitis which often responds to immunosuppression with agents such as cyclophosphamide or mycophenolate. Patients with UIP (such as those with RA) may need a different approach. Biologic therapy as well as aggressive immunomodulation has begun to improve the outlook for such patients in recent years.^36^ The distinction between potentially reversible inflammatory disease (NSIP) and progressive fibrotic lung disease (UIP) is further augmented by the extent of lung involvement.

**Table 1:** Changes in (a) percentages of deaths occurring as a result of ILD, (b) median age at death from ILD, and (c) median survival in those dying from ILD, as related to year of onset in 4 × 7 year cohorts.

*Diagnosis of RA-ILD (by year)*	*Number of Patients*	*Number of Deaths*	*% Dying from ILD*	*Age at Death*	*Survival (Months)*
1988–94	16	14	67%	63 years	33
1995–01	34	22	42%^*^	68 years^*^	36
2002–08	70	26	42%^*^	72 years^*^	50^*^
2009–15	170	18	30%^**^	77 years^**^	80^**^

* *P*<*0.05 and*

** *P*<*0.01*

When considering treatment options, it is important for the clinician to distinguish between drugs that cause harm, those that avoid harm, those that reduce rate of respiratory decline and those that improve function. Latter two may be considered to be of therapeutic value. For many years the therapeutic approach for the management of a wide range of ILD involved a combination of oral steroids and azathioprine. This approach was formally assessed in the PANther trial and found to be associated with a higher mortality than placebo.^37^ The BRILL network also studied the effect of steroid therapy. We found that patients who had taken oral prednisone over a period of more than 3 months were more likely to die during follow-up, despite close matching between the groups of RA-ILD patients on steroids and those not taking them with regard to age, gender, disease duration and extent of ILD, smoking, serology and lung function, with a relative risk (RR) of death from any cause of 1.65 (1.2–2.3; p=0.002) in steroid-treated RA-ILD patients.^38^ Possible confounding by indication was certainly possible as the reasons for selecting patients for steroid therapy were far from clear, but the results were consistent with PANther and suggested that long term steroids may contribute to mortality as a result of the increased risk of infection.

The BRILL network also assessed the effect of disease modifying anti-rheumatic drugs (DMARDs) on outcome in RA-ILD patients. We found no significant effect on mortality associated with the use of any of the usual drugs, including methotrexate, sulphasalazine, leflunomide or hydroxychloroquine. By contrast we also compared outcomes between those patients treated with azathioprine and those who had received mycophenolate, and showed a significant reduction in mortality of RR= 0.65 (0.2–2.0) in those on the latter agent, while azathioprine was associated with a higher than expected mortality, again consistent with results from PANther.^39^

**Figure 2.**
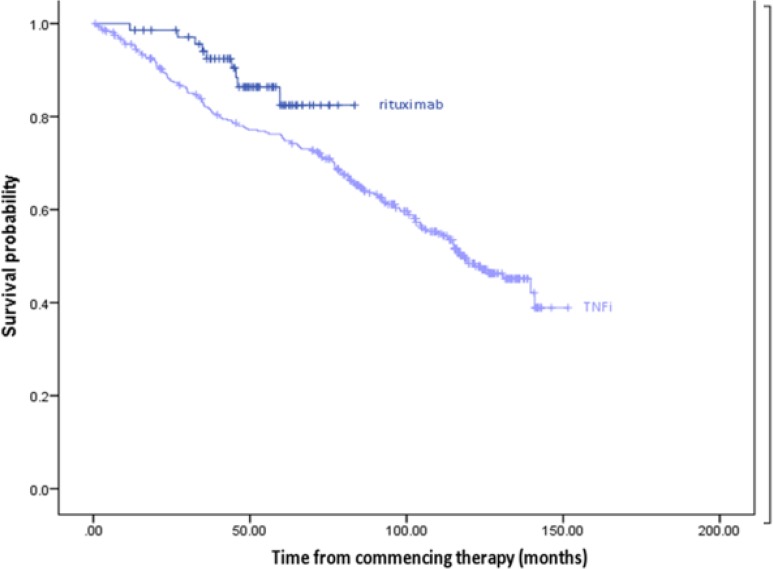
A Kaplan-Meier graph comparing outcomes in survival between patients with RA-ILD on rituximab vs. those on anti-TNF as first choice biologic agent (p=0.02).

All anti-TNF agents have been reported to be associated with acceleration of progression of ILD in some patients,^40^ whereas this is only the case for Rituximab when it had been used in high dose for Haematology patients.^41^ Data for Abatacept and Tocilizumab suggested relative safety but offered little evidence of efficacy in the treatment of RA-ILD. There was interest in the role of B cells in the development of ILD in RA to justify investigating the potential therapeutic role of Rituximab in the treatment of RA-ILD, especially in those patients whose articular disease was sufficient severity to justify biologics. The BRILL network showed better respiratory outcomes for patients with RA-ILD when treated with Rituximab as opposed to anti-TNF therapy (*Table 2*). The British Society for Rheumatology (BSRBR) had collected data across the whole UK on patients with RA-ILD who were treated with different biologics. In total, 377 RA-ILD patients received anti-TNF therapy as their first biologic, while 88 were given Rituximab first line. Mortality at both 3 and 5 years was greater in the anti-TNF group (42 ACR 2016), although there is some data to suggest that this difference became less over time. In Leeds, 56 patients with RA-ILD were treated with Rituximab. They reported that in 68%, ILD either stabilised or improved. Vital capacity and gas transfer also improved overall.^43^ When the data from these sources is combined with that from the BRILL network to produce information on 584 RA-ILD patients over a follow up period of 7 years, there were distinct differences in outcome between the 403 patients treated with anti-TNF and the 181 who received Rituximab. Those on Rituximab had longer overall survival than those on anti-TNF therapy (*Figure 2*).

**Table 2:** Characteristics of patients with RA-ILD and RA controls, and the influence of biologic therapy on all cause and respiratory mortality.

	**RA-ILD**	**anti-TNF**	**Rituximab**	**P value**
Number	290	44	37	-
Median age (years)	65	71	71	0.02
Median RA duration (yr)	10	12	11	NS
Median ILD duration (yr)	5	4	4	NS
Vital capacity (% pred)	80	83	76	NS
Extensive disease (%)	40	52	48	NS
UIP (%)	68	60	77	0.04
All cause mortality (%)	22	31	8	0.03
Respiratory mortality (%)	10	15	4	0.04

When the data on 181 UK Rituximab treated patients was compared with data from 178 age, gender and disease matched patients treated at the Mayo Clinic,^44^ further differences emerged. Three- and five-year survival was significantly greater in the UK at 86% and 82% by comparison with the US cohort where corresponding survival was 75% and 59%, respectively [p=0.002]. This difference may be related to the fact that only small numbers of patients treated at the Mayo received Rituximab, with much larger numbers given anti-TNF. Another potentially important difference between the groups was that immunosuppression comprised azathioprine in the US but was usually mycophenolate in UK treated patients, and this may have also influenced survival rates.^45^

The overall evidence suggests that if a Biologic agent is needed to treat RA in the presence of ILD, anti-TNF agents should be avoided. In those patients who are either smokers or seropositive, we recommend first line therapy with Rituximab, while for seronegative patients, never smokers or those failing to respond to Rituximab, we recommend Abatacept or Tocilizumab. Recently the development of agents for the treatment of fibrotic lung disease has opened the opportunity to transfer the benefits of these agents in idiopathic pulmonary fibrosis (IPF) to those with ILD related to rheumatic disease. Data is being collected across a range of rheumatic disorders with ILD using Nintedanib and Pirfenidone, both of which have been shown to stabilise lung progression in IPF.^46,47^ Other new studies are assessing safety and efficacy of Abatacept and comparing Rituximab with Cyclophosphamide in CTD – ILD and exploring the potential role of Mycophenolate and Rituximab in the treatment of IPF serological subtypes.
